# *De Novo* Large Deletion Leading to Fragile X Syndrome

**DOI:** 10.3389/fgene.2022.884424

**Published:** 2022-05-11

**Authors:** Poonnada Jiraanont, Esther Manor, Nazi Tabatadze, Marwa Zafarullah, Guadalupe Mendoza, Gia Melikishvili, Flora Tassone

**Affiliations:** ^1^ Faculty of Medicine, King Mongkut’s Institute of Technology Ladkrabang, Bangkok, Thailand; ^2^ Faculty of Health Sciences, Ben-Gurion University of the Negev, Beersheba, Israel; ^3^ Genetics Institute, Soroka Medical Center, Beersheba, Israel; ^4^ Department of Pediatrics, MediClub Georgia Medical Center, Tbilisi, Georgia; ^5^ Department of Biochemistry and Molecular Medicine, School of Medicine, University of California, Davis, Sacramento, CA, United States; ^6^ UC Davis MIND Institute, UC Davis Health, Sacramento, CA, United States

**Keywords:** fragile X syndrome, *FMR1* gene, miRNA, *ASFMR1/FMR4* gene, large deletion

## Abstract

Fragile X syndrome (FXS) is the most frequent cause of X-linked inherited intellectual disabilities (ID) and the most frequent monogenic form of autism spectrum disorders. It is caused by an expansion of a CGG trinucleotide repeat located in the 5′UTR of the *FMR1* gene, resulting in the absence of the fragile X mental retardation protein, FMRP. Other mechanisms such as deletions or point mutations of the *FMR1* gene have been described and account for approximately 1% of individuals with FXS. Here, we report a 7-year-old boy with FXS with a *de novo* deletion of approximately 1.1 Mb encompassing several genes, including the *FMR1* and the *ASFMR1* genes, and several miRNAs, whose lack of function could result in the observed proband phenotypes. In addition, we also demonstrate that *FMR4* completely overlaps with *ASFMR1*, and there are no sequencing differences between both transcripts (i.e., *ASFMR1/FMR4* throughout the article).

## Introduction

**FIGURE 1:**
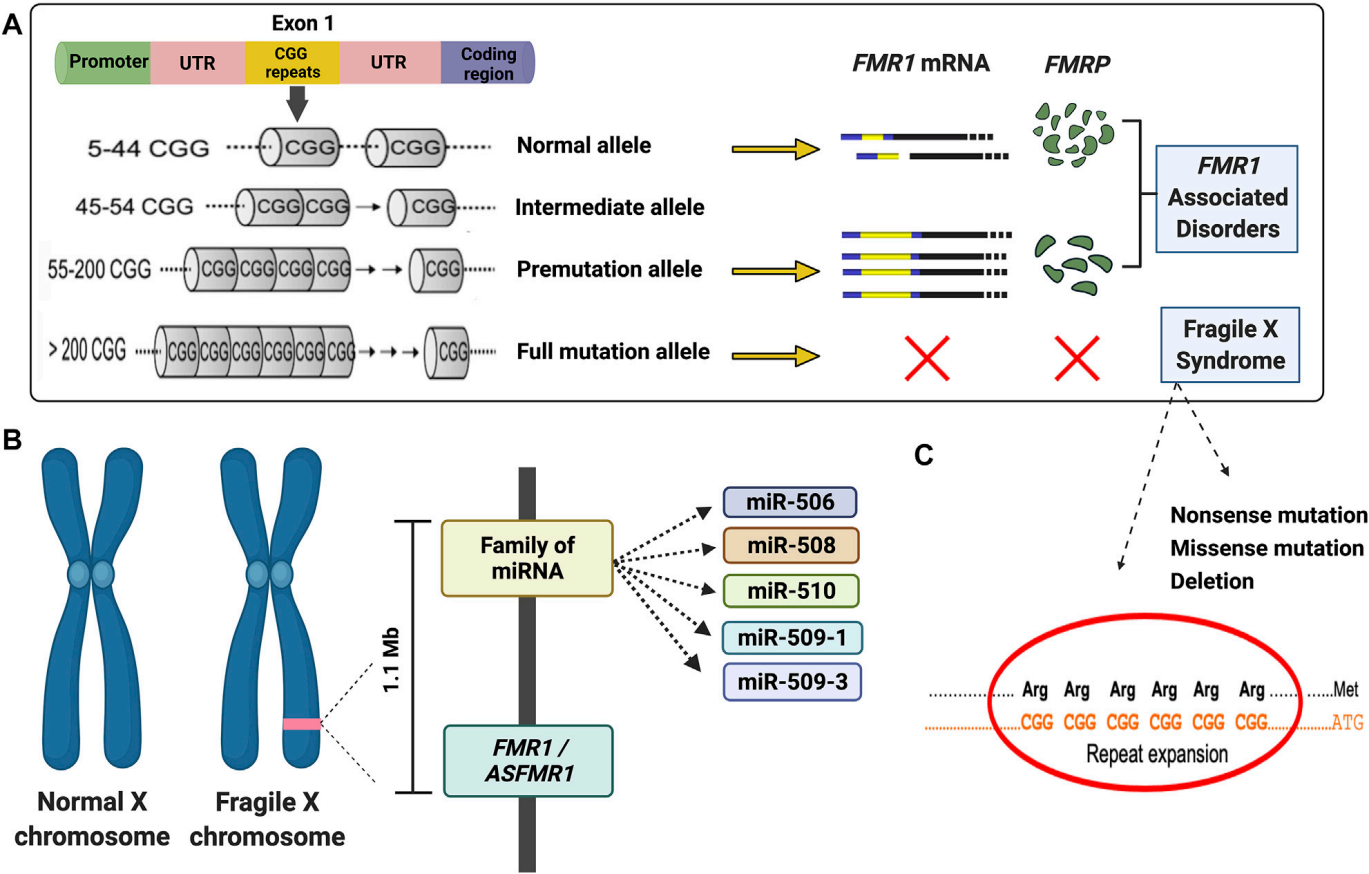
Diagram illustrating the main molecular mechanisms leading to fragile X syndrome and *FMR1*-associated disorders. **(A)** Schematic of molecular causes of fragile X syndrome and *FMR1*-associated disorders. **(B)** Diagram of the deleted region detected in the proband. **(C)** Majority of the fragile X syndrome cases are due to an expansion of the CGG repeat in the 5′UTR of the *FMR1* gene. However, several mutations, including missense, nonsense, and deletion (as demonstrated in the proband) have been reported. A list can be found in the Human Gene Mutation Database for FXS (http://www.hgmd.cf.ac.uk/ac/gene.php?gene=FMR1).

Fragile X syndrome (FXS) is the most prevailing cause of X-linked inherited intellectual disability and autism spectrum disorder (Penagarikano et al., 2007; Rogers et al., 2001). It is caused by a CGG trinucleotide repeat expansion within the 5’ UTR region of the fragile X mental retardation 1 (*FMR1*) gene located on chromosome Xq27.3, which spans approximately 38 kb of genomic sequence (Verkerk et al., 1991; Yu et al., 1991; Fu et al., 1991; Bell et al., 1991). The CGG repeats expand in the female germline during transmission. When the CGG expands to more than 200 repeats, the so-called full mutation, the promoter of *FMR1* becomes hypermethylated and transcriptionally silent, leading to gene inactivation and consequently diminished or lack of expression of the *FMR1* protein, FMRP (Fu et al., 1991; Tassone et al., 2000) (Figure 1A **)**. Individuals with the fragile X premutation (55-200 CGG repeats) are at risk of developing a number of clinical problems falling under the umbrella of *FMR1*-associated disorders. Male individuals with FXS have moderate to severe intellectual impairment, and a behavioral phenotype characterized by repetitive behaviors, social difficulties, poor eye contact, excessive shyness, anxiety, aggression, tactile defensiveness, hyperarousal response to sensory stimuli, tantrums, attention deficits, hyperactivity, impulsivity, self‐injury, stereotypic movements including hand flapping, and perseverative speech. Physical manifestations range from the large forehead, prominent ears, hyperextensible finger joints, and macroorchidism (Hagerman and Hagerman, 2002; Hatton et al., 2002; Reiss and Dant, 2003; Hagerman et al., 2009; Symons et al., 2010; Kidd et al., 2014).

Although the CGG repeat expansion is the main underlying cause of FXS, other mechanisms, including point mutations or deletions, can lead to FXS (Handt et al., 2014; Monaghan et al., 2013; Quartier et al., 2017; Sitzmann et al., 2018) (Figures 1B,C). The deletions in FXS individuals commonly range from hundreds to several millions of base pairs and frequently encompass a portion or the entire sequence of the *FMR1* gene (Coffee et al., 2008). Several reports during the last 3 decades demonstrated *de novo* deletions (Tarleton et al., 1993; Gu et al., 1994; Petek et al., 1999; Luo et al., 2014; Zink et al., 2014; Jorge et al., 2018; Myers et al., 2019) spanning the *FMR1* or both the *FMR1* and *FMR2* gene (Clarke et al., 1992; Wolff et al., 1997; Fengler et al., 2002; Probst et al., 2007), resulting in the loss of FMRP and a range of phenotypes including physical, cognitive and behavioral features, intellectual disabilities (ID), seizures, and obesity, similarly observed in individuals with FXS (Coffee et al., 2008).

The human genome is extensively transcribed and gives rise to various long non-coding RNAs (lncRNAs), defined as RNAs longer than 200 nucleotides and not translated into functional proteins (Fang et al., 2018). Approximately 40% of mammalian lncRNAs are expressed in the brain (Briggs et al., 2015) and play an essential role in neuronal differentiation and regeneration (Perry et al., 2018). They regulate significant biological roles in DNA damage response and cellular senescence (Tsai et al., 2010; Hung et al., 2011; Kotake et al., 2011; Pastori et al., 2015) through modulation of gene expression regulation. In addition, several lncRNAs play a role in brain development, synaptic signaling mechanisms, differentiation of neural cell lineage, and formation of mature neuronal connections (Mercer et al., 2010; Qureshi et al., 2010). Altered expression of lncRNAs has been associated with neurodevelopmental disorders like Prader–Willi syndrome (Statello et al., 2021) and neurodegenerative disorders such as Parkinson’s disease, Huntington’s disease, lateral amyotrophic sclerosis, or Alzheimer’s disease (Wei et al., 2018).

A comprehensive analysis of the transcriptional landscape of the human *FMR1* gene, discovered a decade ago, the *ASFMR1* gene, an lncRNA, a unique antisense transcript, overlapping the CGG repeat region at the *FMR1* locus. Its expression is determined by two promoters that are flanked by CTCF-binding sites: the *FMR1* bidirectional promoter and the other one located in the second intron of the *FMR1* gene, which is the major promoter in premutation cells (Ladd et al., 2007). The *ASFMR1* mRNA, similarly to the *FMR1*, is upregulated in lymphoblastoid cells and peripheral blood leukocytes derived from individuals with the premutation, and it is not expressed in subjects with the *FMR1* full mutation (Ladd et al., 2007; Loesch et al., 2011). In addition, the *ASFMR1* transcript demonstrates premutation-specific alternative splicing suggesting a potential contribution of bidirectional transcription to the clinical phenotypes of the *FMR1*-associated disorders (Al Olaby et al., 2018; Vittal et al., 2018; Zafarullah et al., 2020). Additional studies have revealed the presence of several lncRNAs within the FMR1 gene, including, *FMR4* (Khalil et al., 2008), *FMR5*, and *FMR6* (Pastori et al., 2014). Like the *FMR1* mRNA, *FMR4*, a primate-specific non-coding RNA transcript (∼2.4 kb) residing upstream and sharing a bidirectional promoter with *FMR1*, is silenced in patients with FXS and upregulated in carriers of an *FMR1* premutation allele. It was reported that *FMR4* has an antiapoptotic function in HEK293T and HeLa cells but no effect on *FMR1* gene regulation, suggesting a potential indirect regulatory transcript for *FMR1* (Khalil et al., 2008).

Furthermore, numerous studies have indicated that FMRP acts as a mediator effector of the microRNA (miRNA) pathway by interacting with miRNA and proteins to form RNA-induced silencing complex (RISC) (Caudy et al., 2002; Ishizuka et al., 2002; Jin et al., 2004; Smalheiser and Lugli, 2009; Dionne and Corbin, 2021). Thus, the lack of FMRP in FXS and its role in the miRNA pathway contribute to the abnormal synaptogenesis in FXS and provide one of the mechanisms underlying the fragile X physiopathology (Edbauer et al., 2010; Gong et al., 2015; DeMarco et al., 2019).

Here, we report an FXS case of a seven-year-old boy with a *de novo* deletion of chromosome X of approximately 1.1 Mb encompassing several genes, including the *FMR1* and *ASFMR1* genes and the miR-506 family of miRNAs (Figure 1B). Their absence is likely related to neurodevelopment and his FXS phenotypes. Interestingly, we also demonstrated that *FMR4* completely overlaps with *ASFMR1*, and there are no sequencing differences between both transcripts, therefore named in this study as *ASFMR1/FMR4* gene. The characterization of the *ASFMR1* gene, using SMRT sequencing, identified ∼6-kb *ASFMR1* transcript that overlaps with *FMR4*, demonstrating that *FMR4* and *ASFMR1* are the same lncRNA (*ASFMR1/FMR4*).

## Materials and Methods

### DNA Testing: CGG Sizing

The genomic DNA of the proband, mother, and sisters was isolated from peripheral blood leukocytes (3-5 ml of whole blood) using standard methods (Puregene Kit; Gentra Inc, Minneapolis, MN). PCR and Southern blot analysis were performed to determine the CGG sizing and methylation status. For Southern Blot analysis, 5-10 μg of DNA digested with EcoRI and NruI fixed on a nylon membrane was hybridized with the *FMR1* genomic probe StB12.3, labeled with Dig-11-dUTP by PCR (PCR Dig Synthesis Kit; Roche Diagnostics) following the protocol as previously described (Tassone et al., 2008; Filipovic-Sadic et al., 2010).

### Chromosomal Microarray

CMA was carried out on the proband using GeneChip auto scan 750 K (Affymetrix Santa Clara, United States) following the manufacturer's instructions. A chromosomal analysis was performed using Chromosome Analysis Suite (ChAS®) software (Affymetrix, Santa Clara, United States). The CNVs found in the patient were analyzed in comparison with public databases, including Database of Genomic Variants (DGV), Database of Chromosomal Imbalance and Phenotype in Humans using Ensemble Resources (DECIPHER), and CytoScanHD™ Array Database. Furthermore, CNVs were classified according to their nature, based on (Miller et al., 2010; Battaglia et al., 2013). After the initial Array-CGH result was acquired, informed written consent was obtained from the patient’s mother to draw blood on all available family members for additional investigation. The blood samples were obtained from the patient, his mother, his sisters, and his maternal uncle for further analysis.

### Sequencing of the *ASFMR1* mRNA

The total RNA was isolated from postmortem brains derived from three normal individuals in a clean and RNA designated area using TRIzol reagent (Invitrogen, Carlsbad, CA), followed by quantification via a Qubit fluorometer (Invitrogen, Waltham, MA, United States) and quality control by measurements of the RNA integrity number utilizing an Agilent 2100 Bioanalyzer system.

The genomic sequence data of 2198-bp *FMR4* at locus FJ887036 and 2942-bp *ASFMR1* at locus EU048204 were obtained from the UCSC website (http://genome.ucsc.edu/). The *ASFMR1* transcripts were identified by PacBio iso-sequencing from isolated total RNA from three brain samples. Then 1 ug of total RNA was reverse transcribed using the Clontech SMARTer cDNA synthesis kit and six samples specific barcoded oligo dT (with PacBio 16mer barcode sequences). Three reverse transcription (RT) reactions were processed in parallel for each sample. PCR optimization was used to determine the optimal amplification cycle number for the downstream large-scale PCRs. A single primer (primer IIA from the Clontech SMARTer kit 5′ AAG CAG TGG TAT CAA CGC AGA GTA C 3′) was used for all PCRs post-RT. Large-scale PCR products were purified separately with 1X AMPure PB beads, and the bioanalyzer was used for QC. An equimolar two pools of 3-plex barcoded cDNA library were input into the probe-based capture with a custom-designed *ASFMR1* gene panel. A SMRTBell library was constructed using captured and reamplified cDNA. One SMRT Cell 1M (20 h movie) was sequenced on the PacBio Sequel platform using 2.0 chemistry. The isoform sequencing analysis was performed using the IsoSeq3 application in the PacBio SMRT Analysis v6.0.0.

## Results

### Clinical History

The patient we present in this study is a 7-year-old boy with moderate intellectual disability (IQ = 41, WISC-V), attention deficit, and hyperactivity. He was a full-term male infant and third child of nonconsanguineous parents, born after a normal pregnancy. His birth weight was 4,500 g, and there were no perinatal problems. His developmental milestones included sitting at 6 months, walking at 21 months, using words at 17 months, and phrases by 3.5 years of age.

On physical examination at age 7 years, his weight was 29 kg (92nd percentile), height was 125 cm (45th percentile), and head circumference was 52.5 cm (64th percentile). The HEENT (head, eyes, ears, nose, and throat) examination was normal. The neurological examination findings were normal. Other characteristics included a long face, prominent forehead, large and protruding ears, normal palate, long palpebral fissures, epicanthal folds, flat nasal bridge, pectus excavatum, macroorchidism, and no joint hypermobility. There are no cardiovascular abnormalities, and echocardiography showed normal results. Overall, the patient is physically a well-developed boy with no significant anomalies.

He hears normally and follows simple commands. He has an acceptable motor delay and deficits in visual analyses and synthesis. He can eat independently regarding daily living skills and uses the toilet but cannot get dressed without help. The child exhibits hand biting and hand flapping when excited, his mood is generally good, and he shows no aggressive behavior toward others but sometimes he is very anxious. The parents noted excessive shyness, and he consistently avoids eye contact by covering his eyes with his hands, although he is exceptionally social and can interact with others. His early behavior included ADHD. He is frequently irritable and hyperactive at home, exhibits concentration problems, short attention span, distractibility, and impulsiveness. He has learning problems, especially difficulty with mathematics.

**FIGURE 2:**
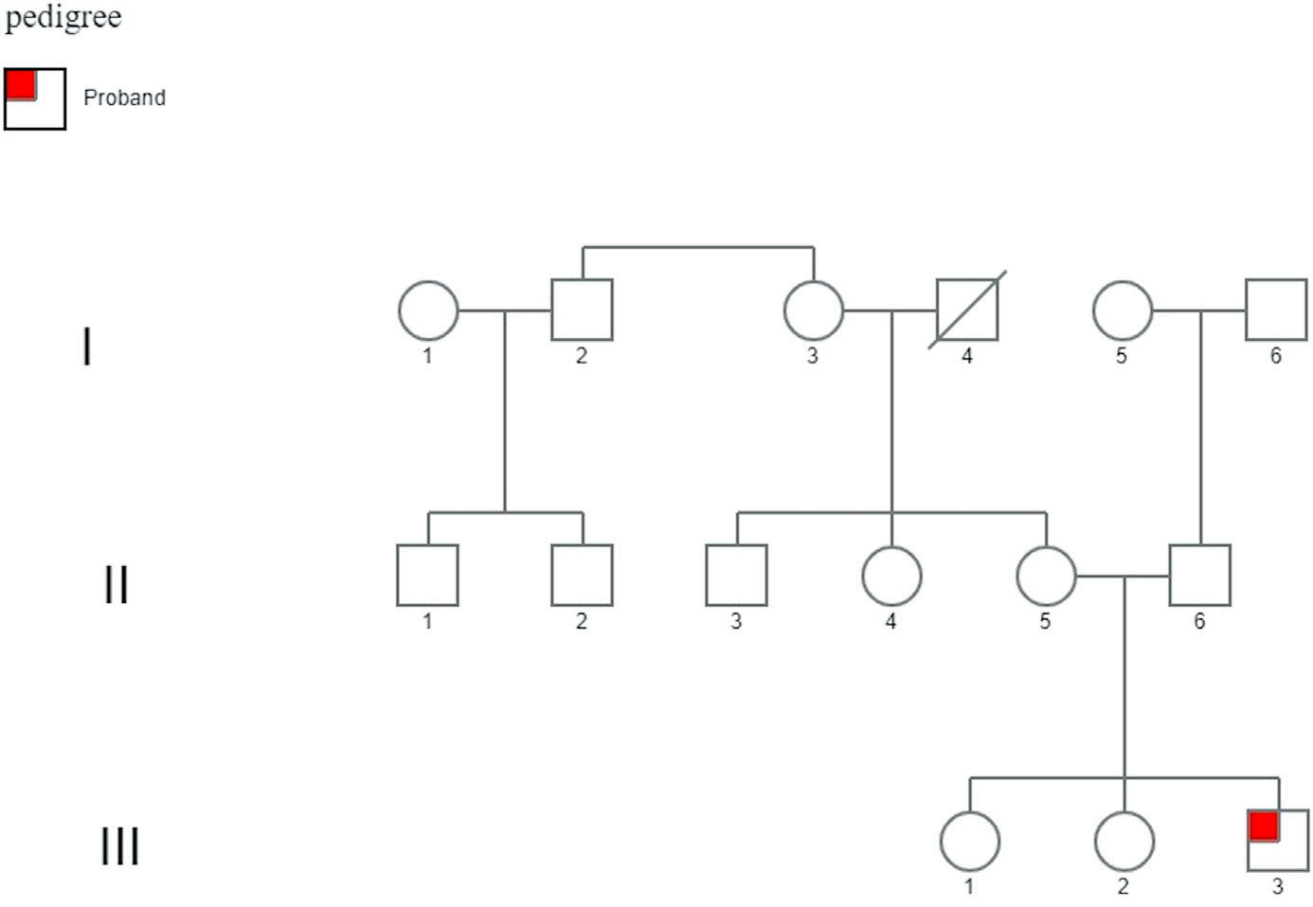
Family pedigree. His family history demonstrates two healthy 16- and 14-year-old sisters. The maternal uncle (II-3) displayed mild to moderate intellectual disability, and the maternal aunt (II-4) displayed psychiatric problems (depressive disorder) and ovarian cysts, unrelated to a mutation in the *FMR1* gene.

The proband has a healthy mother and healthy 16 and 14-years old sisters; the maternal uncle has mild to moderate intellectual disability, while the maternal aunt has depressive disorders and ovarian cysts **(** Figure 2 **).**

### Molecular Testing: CGH Array and FXS Diagnostic Testing

At the age of 7 years, the proband underwent array-CGH on DNA extracted from peripheral blood cells (244 K kit, Agilent Technologies, Santa Clara, CA, United States), which revealed a deletion of approximately 1.1 Mb located on Xq27.3 (NC_000,023.10: g 145,877,075-147,047,871) encompassing several genes and miRNAs, including the entire *FMR1* and *ASFMR1/FMR4* genes and microRNAs (miRNAs) such miR506, miR508, miR509-1, miR509-3, and miR510. Molecular DNA testing for FXS, carried out by PCR and Southern blot analysis, showed no PCR amplification and lack of hybridization with the *FMR1* specific probe in the proband, indicating the presence of a deletion of the *FMR1* gene **(** Supplementary Figure S1). The mother was not a carrier of the *FMR1* premutation as determined by Southern Blot and PCR analysis.

### The lncRNA, *ASFMR1*, and *FMR4* Are the Same Transcripts

**FIGURE 3:**
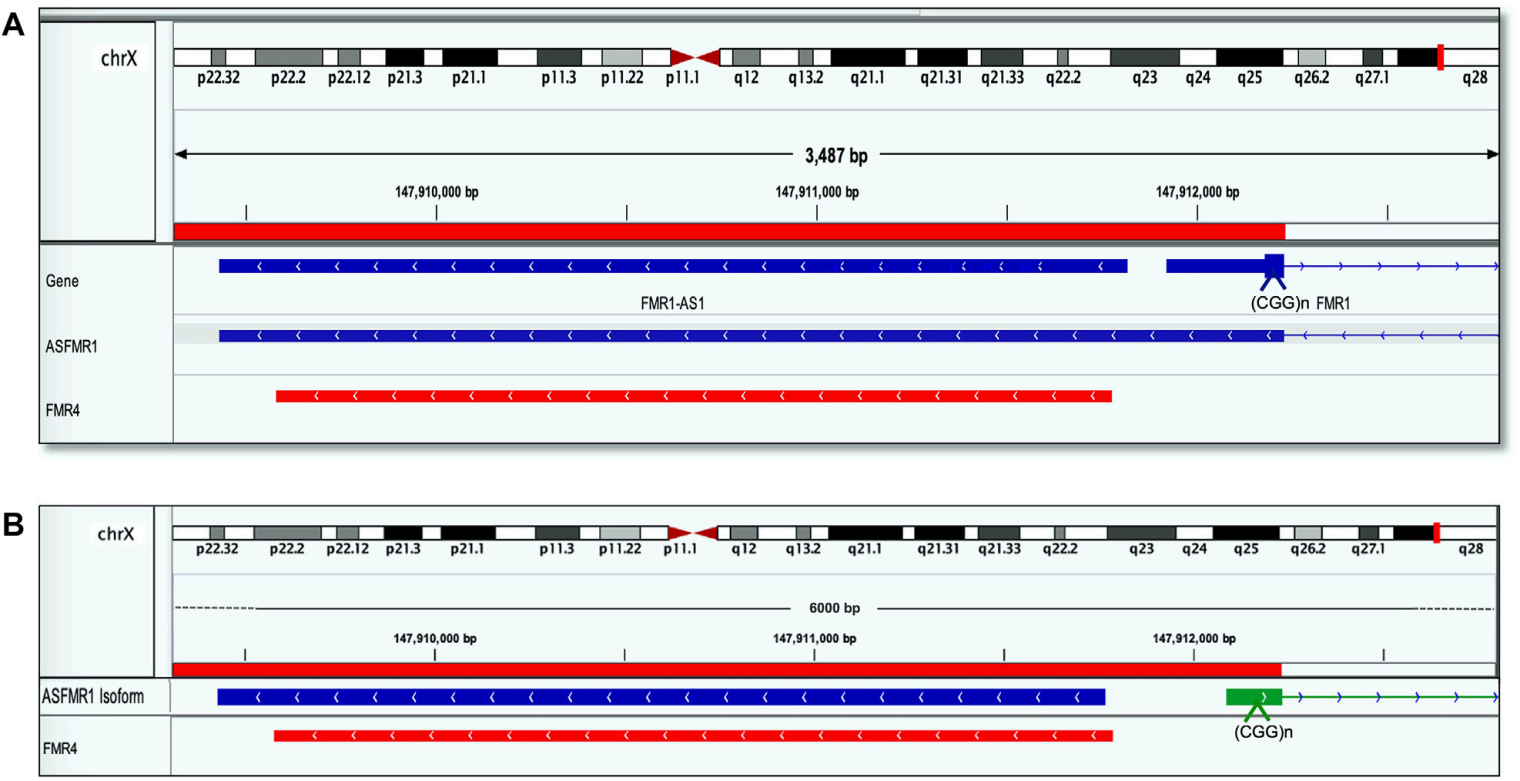
*FMR4* overlaps with *ASFMR1*. The figure shows a visualization of sequence alignment in Integrative Genomic Viewer (IGV) at 3487 base-pair resolution on chromosome X in human genome assembly GRCh38 (hg38). **(A)** *ASFMR1* and *FMR1* genes from the human genome assembly are shown (the top of the three tracks are highlighted in blue). The middle track is the *ASFMR1* transcript reported by (Ladd et al., 2007) overlapping with the *FMR1* gene (going in the opposite direction). For the *FMR1* gene, only exon 1 (containing the CGG repeat) and intron 1 (thin blue line) are shown. The bottom track (highlighted in red) shows the sequence of the *FMR4* reported by (Khalil et al., 2008). The location of the CGG repeat in the *FMR1* is indicated (CCG in the antisense direction). **(B)** Diagram shows the overlap between the two transcripts *ASFMR1* (highlighted in blue) and *FMR4* (highlighted in red) on a zoom image of the 6-kb *ASFMR1* identified transcripts. The overlap demonstrates the sequencing identity of the two transcripts. The location of the CGG repeat in the *FMR1* is indicated (CCG in the antisense direction).

The sequence identity of *ASFMR1/FMR4* using the Integrative Genomic Viewer (IGV) was obtained by blasting the transcriptomic sequences reported in the UCSC human genome database. Interestingly, we identify the *ASFMR1* transcript in our library data set, and by sequencing comparison, we confirmed that no genetic sequence differences exist between the two transcripts **(** Figure 3A **)**, as one of the hypotheses, proposed by Khalil et al. (2008). The complete sequence overlaps between the two genes (*ASFMR1/FMR4*) are shown inFigure 3B.

## Discussion

We report a case of FXS caused by a *de novo* deletion spanning a 1.1 Mb of DNA comprising the *FMR1* and the *ASFMR1* genes, and the miR-506 family of miRNAs. Importantly, we verified the overlap of the *ASFMR1/FMR4* genes, previously identified which are therefore the same transcripts.

In 2007, 1 year prior to the discovery of the *FMR4* lncRNA, Ladd (Ladd et al., 2007) identified the novel *ASFMR1* (antisense fragile X mental retardation 1) gene, highly expressed in the brain and kidney but hardly noticeable in the heart. Similarly, to the *FMR1* gene, the novel *ASFMR1* transcript was upregulated in premutation alleles but repressed in full mutations and exhibited premutation-specific alternative splicing (Ladd et al., 2007; Zafarullah et al., 2020). One of the alternative splicing isoforms, *ASFMR1* mRNAs Iso131 bp, positively correlates with the CGG repeat number and could distinguish between carriers of the premutation and controls, and its expression levels in premutation carriers, with and without FXTAS, were significantly different from controls (Vittal et al., 2018; Zafarullah et al., 2020).

In 2008, Khalil and his colleagues reported on the identification of *FMR4*, a novel 2.4-kb lncRNA, which transcribed in an antisense direction upstream of the *FMR1* gene. The authors showed that the *FMR4* transcript was highly expressed in the heart and kidney of human fetal tissues and in human adult brains including the hippocampus, frontal cortex, and cerebellum. Thus, similarly to the *FMR1* gene, the *ASFMR1/FMR4* mRNA expression is silenced in the brain of full mutations but highly expressed in premutation carriers. In addition, *ASFMR1/FMR4* demonstrated an antiapoptotic function *in vitro* suggesting it may promote human cell proliferation (Khalil et al., 2008). Further study revealed that the *ASFMR1/FMR4* transcript suppresses MBD4 (methyl-CpG-binding domain protein 4), which is a translational repressor, pivotal for DNA mismatch repair, inhibition of transcription, and regulation of apoptosis, in trans-activity (Yakovlev et al., 2017). Interestingly, *ASFMR1/FMR4* expression is significantly reduced, while both *FMR1* and *MBD4* expressions are increased, in differentiating human neural precursor cells proposing a role for *ASFMR1/FMR4* as a gene-regulatory lncRNA in normal development (Peschansky et al., 2015). In addition, overexpression and silencing of *ASFMR1/FMR4* can induce genome-wide alteration in histone methylation of several genes implicating developmental or neurophysiological roles (Peschansky et al., 2016). These findings suggest that *ASFMR1/FMR4* may regulate neurodevelopment, and its level of expression could influence the pathogenesis of *FMR1*-associated disorders.

In this study, we confirmed the complete sequence overlaps between the two transcripts, the *ASFMR1* mRNA and the *FMR4* mRNA, which is not surprising given the similar characteristics and qualities. In addition to the different reported lengths, the *ASFMR1/FMR4* transcript is polyadenylated, and expressed highly in the brains of premutation carriers but suppressed in full mutation individuals. Notably, evidence demonstrated that the *ASFMR1/FMR4* gene plays a role in neurodevelopment as it is involved in early neural differentiation and emerged as a promising biomarker for FXTAS, a neurodegenerative disorder (Peschansky et al., 2016; Vittal et al., 2018). Taken together, the deletion encompassing *the ASFMR1/FMR4* region may influence the neurodevelopment and neuropathogenesis observed in the proband.

Furthermore, the deletion present in the proband also included a panel of miR-506 family miRNAs consisting of miR506, miR508, miR509-1, miR509-3, and miR510, all belonging to the “fragile-X miRNA (FX-MIR)” cluster (Ramaiah et al., 2019). miRNAs are crucial for various critical functions of cellular differentiation and homeostasis, as they regulate the expression of hundreds of mRNAs resulting in diverse pathologies, including cancer, neural disorders, and infertility (Greenberg and Soreq, 2014; Khazaie and Nasr Esfahani, 2014; Bracken et al., 2016). miRNAs are approximately 22-nucleotide-long small RNAs that regulate the gene expression at post-transcriptional levels by binding with target mRNAs like FMRP and Argonaute protein incorporated into the RNA-induced silencing complex (RISC) to silence their target mRNAs (Filipowicz et al., 2008; Edbauer et al., 2010; Santhekadur and Kumar, 2020). The FX-MIR is a member of an X chromosome-linked miRNA cluster initially determined in primate testis and found well conserved among placental mammals (Bentwich et al., 2005; Zhang et al., 2007; Li et al., 2010). The FX-MIR consists of 22 miRNAs, located and transcribed from five large clusters on the X chromosome and encompassing approximately 62-kb region. One of these clusters is adjacent to *FMR1*, which suggests the possibility that the FX-MIR miRNAs might target the *FMR1* (Fromm et al., 2015; Wang et al., 2020). Most of them are primarily expressed in the human testis and brain, in which *FMR1*, their target, is also highly expressed in these two organs. Accordingly, the FX-MIR cluster may play a role in gene expression in both tissues since it can lead to silencing and the consequent absence of FMRP (Tamanini et al., 1997; Peier et al., 2000). Furthermore, the loss of function of FMRP demonstrated deterioration of synaptic formation and plasticity in the central nervous system and macroorchidism by overproduction of sertoli cells in the testes (Slegtenhorst-Eegdeman et al., 1998; Terracciano et al., 2005). A subset of the FX-MIR cluster has increased expression levels significantly in FXS NPCs and neurons, in which miR-509-3p and miR-890 were upregulated by approximately 78-fold and 106-fold, respectively, compared to the control. Thus, dysregulation of specific miRNAs may play a vital role in FXS (Ramaiah et al., 2019). Consistently with these findings, our proband exhibited moderate ID and ADHD, and macroorchidism, partly due to deletion of the FX-MIR cluster.

Albeit the prevalent mechanism of FXS is the presence of hypermethylation of the CGG repeat tract and the upstream CpG island in the promoter region leading to transcriptional silencing (Bell et al., 1991; Verkerk et al., 1991), several reports involving point mutations and deletions, either small or large, have been described (Sitzmann et al., 2018; Saldarriaga et al., 2020; Erbs et al., 2021). The deletions affecting the *FMR1* gene account for the second most common cause of FXS, although they are rare (Coffee et al., 2009). Small deletions (<10 kb) are consistently concomitant with full mutation (>200 CGG repeats) or premutation (55-200 CGG repeats) alleles and happen during the transmission of a premutation allele from mother to offspring (Hammond et al., 1997; Han et al., 2006). Rather, large deletions (>10 kb) result in meiotic or mitotic ectopic recombination in which crossing over occurs at non-homologous loci (Coffee et al., 2008). Individuals with large deletions affecting the *FMR1* gene usually display typical features of FXS, including seizure and obesity and if the deletions span over adjacent genes, they may present with additional manifestations (Moore et al., 1999).

A review of the current literature found six male individuals with *de novo* large deletion (>10 kb) as their mothers did not carry either a premutation or a full mutation allele, or a deleted allele and index cases did not have an expansion of CGG repeat but only large deletions harboring the *FMR1* gene and contiguous DNA sequences (Wöhrle et al., 1992; Tarleton et al., 1993; Gu et al., 1994; Hirst et al., 1995; Nagamani et al., 2012; Jiraanont et al., 2016). The reported deletions encompassing the *FMR1* gene range from 35 kb to 3 Mb; they all have typical features of FXS involving ID from moderate to severe and developmental delay (DD) including motor and language development at various severities. Regarding physical anomalies, four out of seven cases, including our case, had dysmorphic features including macroorchidism and shawl scrotum, and two cases presented with macrocephaly. Interestingly, three of them had vision-related problems including epicanthus inversus, esotropia, and our case presents with mild deficits in visual-related cognitive abilities. Three cases had neurodevelopmental disorders, including autistic behaviors and attention and concentration problems. This evidence strongly supports that the development of typical functions of cognition and behavior are regulated by *FMR1*.

Our patient manifested typical FXS characteristics with distinct facial features, macroorchidism, and moderate ID with ADHD. However, he is a well-developed physical boy with good social interaction and delay in fine motor and subtle visual deficits. To date, there are only two studies mentioning *de novo* FXS cases with gain and loss CNV of the *ASFMR1/FMR4* gene (Nagamani et al., 2012; Vengoechea et al., 2012). Our colleagues reported a 4-year-old boy with 86-kb microduplication of the *FMR1* and of the *ASFMR1/FMR4* genes, together with a 363-kb duplication on 1q44 and a 168-kb deletion on 4p15.31 inherited from a healthy father. The boy presented with myoclonic seizures and later developed absence seizures, persistent speech, and fine motor delay. He also had signs and symptoms of hyperactivity (Vengoechea et al., 2012). (Nagamani et al., 2012) reported a 5-year-old boy with a deletion genomic region containing the *FMR1* and the *ASFMR1/FMR4* genes. This boy had relatively macrocephaly and large ears, epicanthus inversus, and bilateral trigones of the lateral ventricles. He could not speak at 4-years of age indicating significant language delay. Our case phenotypes are more like a boy with microduplication of the *FMR1* and of the *ASFMR1/FMR4* genes with a relatively milder phenotype, fine motor delay and ADHD although this boy did not have distinctive facial features and macroorchidism, as in our case. The author suggested that either a loss or a gain copy number of the *FMR1* gene, which is tightly regulated, is essential for the normal development of neurocognitive structures and functions, and can lead to neurodevelopmental disorders (Nagamani et al., 2012). Finally, we report that the deletion observed in the proband is due to a *de novo* deletion, as the two probands’ sisters, the mother and maternal uncle do not have the deletion (Supplementary Figure 1). However, although unlikely, given the two probands’ normal sisters, we cannot completely exclude the possibility of germinal mosaicism in his mother.

Importantly, this study, suggested a complex transcription within the FMR1 locus, and further advanced investigation would be needed to determine the exact nature and function of all the transcripts, and their relevance to the FMR1-associated disorders. An Mb deletion encompassing the *FMR1*, *ASFMR1/FMR4* genes, and FX-MIR cluster detected in the proband may provoke the observed clinical phenotype including intellectual disability, attention-deficit hyperactivity disorder, distinct facial features, and macroorchidism, which are typical characteristics exhibited in individuals with FXS. Importantly, we verified that the *ASFMR1/FMR4* is the same genes.

## Data Availability

The datasets for this article are not publicly available due to concerns regarding participant/patient anonymity. Request to access the datasets should be directed to the corresponding author.

## Supplementary Material

Supplementary Figure S1 Southern blot analysis of genomic DNA isolated from a negative control (lane 1, normal female) and from a positive control (lane 5, full mutation male). Lane 2 shows lack of hybridization to the *FMR1*-specific probe Stb 12.3 in the proband’s DNA indicating the presence of a deletion. Lanes 2 and 3 show a normal band patter in the proband’s mother and in the maternal uncle, respectively. One-kilobyte ladder is shown in lane M. The normal unmethylated band (2.8 kb) and normal methylated band (5.2 kb) in a female individual are shown on the left side. PCR analysis using *FMR1* specific primers did not yield any amplicons (data not showed).

## References

[B1] Al Olaby R. R. Tang H.-T. Durbin-Johnson B. Schneider A. Hessl D. Rivera S. M. (2018). Assessment of Molecular Measures in Non-FXTAS Male Premutation Carriers. Front. Genet. 9, 302. 10.3389/fgene.2018.00302 30186307 PMC6113865

[B2] Battaglia A. Doccini V. Bernardini L. Novelli A. Loddo S. Capalbo A. (2013). Confirmation of Chromosomal Microarray as a First-Tier Clinical Diagnostic Test for Individuals with Developmental Delay, Intellectual Disability, Autism Spectrum Disorders and Dysmorphic Features. Eur. J. Paediatric Neurol. 17, 589–599. 10.1016/j.ejpn.2013.04.010 23711909

[B3] Bell M. V. Hirst M. C. Nakahori Y. MacKinnon R. N. Roche A. Flint T. J. (1991). Physical Mapping across the Fragile X: Hypermethylation and Clinical Expression of the Fragile X Syndrome. Cell 64, 861–866. 10.1016/0092-8674(91)90514-y 1997211

[B4] Bentwich I. Avniel A. Karov Y. Aharonov R. Gilad S. Barad O. (2005). Identification of Hundreds of Conserved and Nonconserved Human microRNAs. Nat. Genet. 37, 766–770. 10.1038/ng1590 15965474

[B5] Bracken C. P. Scott H. S. Goodall G. J. (2016). A Network-Biology Perspective of microRNA Function and Dysfunction in Cancer. Nat. Rev. Genet. 17, 719–732. 10.1038/nrg.2016.134 27795564

[B6] Briggs J. A. Wolvetang E. J. Mattick J. S. Rinn J. L. Barry G. (2015). Mechanisms of Long Non-coding RNAs in Mammalian Nervous System Development, Plasticity, Disease, and Evolution. Neuron 88, 861–877. 10.1016/j.neuron.2015.09.045 26637795

[B7] Caudy A. A. Myers M. Hannon G. J. Hammond S. M. (2002). Fragile X-Related Protein and VIG Associate with the RNA Interference Machinery. Genes Dev. 16, 2491–2496. 10.1101/gad.1025202 12368260 PMC187452

[B8] Clarke J. T. Wilson P. J. Morris C. P. Hopwood J. J. Richards R. I. Sutherland G. R. (1992). Characterization of a Deletion at Xq27-Q28 Associated with Unbalanced Inactivation of the Nonmutant X Chromosome. Am. J. Hum. Genet. 51, 316–322. 1642233 PMC1682679

[B9] Coffee B. Ikeda M. Budimirovic D. B. Hjelm L. N. Kaufmann W. E. Warren S. T. (2008). MosaicFMR1 Deletion Causes Fragile X Syndrome and Can lead to Molecular Misdiagnosis: A Case Report and Review of the Literature. Am. J. Med. Genet. 146A, 1358–1367. 10.1002/ajmg.a.32261 18412117 PMC2697959

[B10] Coffee B. Keith K. Albizua I. Malone T. Mowrey J. Sherman S. L. (2009). Incidence of Fragile X Syndrome by Newborn Screening for Methylated FMR1 DNA. Am. J. Hum. Genet. 85, 503–514. 10.1016/j.ajhg.2009.09.007 19804849 PMC2756550

[B11] DeMarco B. Stefanovic S. Williams A. Moss K. R. Anderson B. R. Bassell G. J. (2019). FMRP - G-Quadruplex mRNA - miR-125a Interactions: Implications for miR-125a Mediated Translation Regulation of PSD-95 mRNA. PLoS One 14, e0217275. 10.1371/journal.pone.0217275 31112584 PMC6529005

[B12] Dionne O. Corbin F. (2021). An "Omic" Overview of Fragile X Syndrome. Biology 10, 433. 10.3390/biology10050433 34068266 PMC8153138

[B13] Edbauer D. Neilson J. R. Foster K. A. Wang C.-F. Seeburg D. P. Batterton M. N. (2010). Regulation of Synaptic Structure and Function by FMRP-Associated MicroRNAs miR-125b and miR-132. Neuron 65, 373–384. 10.1016/j.neuron.2010.01.005 20159450 PMC5018398

[B14] Erbs E. Fenger-Grøn J. Jacobsen C. M. Lildballe D. L. Rasmussen M. (2021). Spontaneous rescue of a FMR1 Repeat Expansion and Review of Deletions in the FMR1 Non-coding Region. Eur. J. Med. Genet. 64, 104244. 10.1016/j.ejmg.2021.104244 34022415

[B15] Fang S. Zhang L. Guo J. Niu Y. Wu Y. Li H. (2018). NONCODEV5: a Comprehensive Annotation Database for Long Non-coding RNAs. Nucleic Acids Res. 46, D308–d314. 10.1093/nar/gkx1107 29140524 PMC5753287

[B16] Fengler S. Fuchs S. König R. Arnemann J. (2002). Mosaicism for FMR1 and FMR2 Deletion: a New Case. J. Med. Genet. 39, 200–201. 10.1136/jmg.39.3.200 11897824 PMC1735074

[B17] Filipovic-Sadic S. Sah S. Chen L. Krosting J. Sekinger E. Zhang W. (2010). A Novel FMR1 PCR Method for the Routine Detection of Low Abundance Expanded Alleles and Full Mutations in Fragile X Syndrome. Clin. Chem. 56, 399–408. 10.1373/clinchem.2009.136101 20056738 PMC4031651

[B18] Filipowicz W. Bhattacharyya S. N. Sonenberg N. (2008). Mechanisms of post-transcriptional Regulation by microRNAs: Are the Answers in Sight? Nat. Rev. Genet. 9, 102–114. 10.1038/nrg2290 18197166

[B19] Fromm B. Billipp T. Peck L. E. Johansen M. Tarver J. E. King B. L. (2015). A Uniform System for the Annotation of Vertebrate microRNA Genes and the Evolution of the Human microRNAome. Annu. Rev. Genet. 49, 213–242. 10.1146/annurev-genet-120213-092023 26473382 PMC4743252

[B20] Fu Y. H. Kuhl D. P. Pizzuti A. Pieretti M. Sutcliffe J. S. Richards S. (1991). Variation of the CGG Repeat at the Fragile X Site Results in Genetic Instability: Resolution of the Sherman Paradox. Cell 67, 1047–1058. 10.1016/0092-8674(91)90283-5 1760838

[B21] Gong X. Wang Y. Zeng J. Li S. Luo Y. (2015). Computational Identification and Experimental Validation of MicroRNAs Binding to the Fragile X Syndrome Gene Fmr1. Neurochem. Res. 40, 109–117. 10.1007/s11064-014-1471-3 25376939

[B22] Greenberg D. Soreq H. (2014). MicroRNA Therapeutics in Neurological Disease. Cpd 20, 6022–6027. 10.2174/1381612820666140314151924 24641229

[B23] Gu Y. Lugenbeel K. A. Vockley J. G. Grody W. W. Nelson D. L. (1994). A De Novo Deletion in FMR1 in a Patient with Developmental Delay. Hum. Mol. Genet. 3, 1705–1706. 10.1093/hmg/3.9.1705 7530551

[B24] Hagerman R. J. Berry-Kravis E. Kaufmann W. E. Ono M. Y. Tartaglia N. Lachiewicz A. (2009). Advances in the Treatment of Fragile X Syndrome. Pediatrics 123, 378–390. 10.1542/peds.2008-0317 19117905 PMC2888470

[B25] Hagerman R. J. Hagerman P. J. (2002). The Fragile X Premutation: into the Phenotypic Fold. Curr. Opin. Genet. Develop. 12, 278–283. 10.1016/s0959-437x(02)00299-x 12076670

[B26] Hammond L. S. Macias M. M. Tarleton J. C. Pai G. S. (1997). Fragile X Syndrome and Deletions in FMR1: New Case and Review of the Literature. Am. J. Med. Genet. 72, 430–434. 10.1002/(sici)1096-8628(19971112)72:4<430:aid-ajmg11>3.0.co;2-s 9375726

[B27] Han X. D. Powell B. R. Phalin J. L. Chehab F. F. (2006). Mosaicism for a Full Mutation, Premutation, and Deletion of the CGG Repeats Results in 22% FMRP and Elevated FMR1 mRNA Levels in a High-Functioning Fragile X Male. Am. J. Med. Genet. A. 140, 1463–1471. 10.1002/ajmg.a.31291 16761284

[B28] Handt M. Epplen A. Hoffjan S. Mese K. Epplen J. T. Dekomien G. (2014). Point Mutation Frequency in the FMR1 Gene as Revealed by Fragile X Syndrome Screening. Mol. Cell Probes 28, 279–283. 10.1016/j.mcp.2014.08.003 25171808

[B29] Hatton D. D. Hooper S. R. Bailey D. B. Skinner M. L. Sullivan K. M. Wheeler A. (2002). Problem Behavior in Boys with Fragile X Syndrome. Am. J. Med. Genet. 108, 105–116. 10.1002/ajmg.10216 11857559

[B30] Hirst M. Grewal P. Flannery A. Slatter R. Maher E. Barton D. (1995). Two New Cases of FMR1 Deletion Associated with Mental Impairment. Am. J. Hum. Genet. 56, 67–74. 7825604 PMC1801332

[B31] Hung T. Wang Y. Lin M. F. Koegel A. K. Kotake Y. Grant G. D. (2011). Extensive and Coordinated Transcription of Noncoding RNAs within Cell-Cycle Promoters. Nat. Genet. 43, 621–629. 10.1038/ng.848 21642992 PMC3652667

[B32] Ishizuka A. Siomi M. C. Siomi H. (2002). A Drosophila Fragile X Protein Interacts with Components of RNAi and Ribosomal Proteins. Genes Dev. 16, 2497–2508. 10.1101/gad.1022002 12368261 PMC187455

[B33] Jin P. Zarnescu D. C. Ceman S. Nakamoto M. Mowrey J. Jongens T. A. (2004). Biochemical and Genetic Interaction between the Fragile X Mental Retardation Protein and the microRNA Pathway. Nat. Neurosci. 7, 113–117. 10.1038/nn1174 14703574

[B34] Jiraanont P. Hagerman R. J. Neri G. Zollino M. Murdolo M. Tassone F. (2016). Germinal Mosaicism for a Deletion of the FMR1 Gene Leading to Fragile X Syndrome. Eur. J. Med. Genet. 59, 459–462. 10.1016/j.ejmg.2016.08.009 27546052

[B35] Jorge P. Garcia E. Gonçalves A. Marques I. Maia N. Rodrigues B. (2018). Classical Fragile-X Phenotype in a Female Infant Disclosed by Comprehensive Genomic Studies. BMC Med. Genet. 19, 74. 10.1186/s12881-018-0589-6 29747568 PMC5946481

[B36] Khalil A. M. Faghihi M. A. Modarresi F. Brothers S. P. Wahlestedt C. (2008). A Novel RNA Transcript with Antiapoptotic Function Is Silenced in Fragile X Syndrome. PLoS One 3, e1486. 10.1371/journal.pone.0001486 18213394 PMC2194623

[B37] Khazaie Y. Nasr Esfahani M. H. (2014). MicroRNA and Male Infertility: A Potential for Diagnosis. Int. J. Fertil. Steril 8, 113–118. 25083174 PMC4107683

[B38] Kidd S. A. Lachiewicz A. Barbouth D. Blitz R. K. Delahunty C. McBrien D. (2014). Fragile X Syndrome: a Review of Associated Medical Problems. Pediatrics 134, 995–1005. 10.1542/peds.2013-4301 25287458

[B39] Kotake Y. Nakagawa T. Kitagawa K. Suzuki S. Liu N. Kitagawa M. (2011). Long Non-coding RNA ANRIL Is Required for the PRC2 Recruitment to and Silencing of p15INK4B Tumor Suppressor Gene. Oncogene 30, 1956–1962. 10.1038/onc.2010.568 21151178 PMC3230933

[B40] Ladd P. D. Smith L. E. Rabaia N. A. Moore J. M. Georges S. A. Hansen R. S. (2007). An Antisense Transcript Spanning the CGG Repeat Region of FMR1 Is Upregulated in Premutation Carriers but Silenced in Full Mutation Individuals. Hum. Mol. Genet. 16, 3174–3187. 10.1093/hmg/ddm293 17921506

[B41] Li J. Liu Y. Dong D. Zhang Z. (2010). Evolution of an X-Linked Primate-specific Micro RNA Cluster. Mol. Biol. Evol. 27, 671–683. 10.1093/molbev/msp284 19933172

[B42] Loesch D. Z. Godler D. E. Evans A. Bui Q. M. Gehling F. Kotschet K. E. (2011). Evidence for the Toxicity of Bidirectional Transcripts and Mitochondrial Dysfunction in Blood Associated with Small CGG Expansions in the FMR1 Gene in Patients with Parkinsonism. Genet. Med. 13, 392–399. 10.1097/GIM.0b013e3182064362 21270637 PMC4022481

[B43] Luo S. Huang W. Xia Q. Xia Y. Du Q. Wu L. (2014). Cryptic FMR1 Mosaic Deletion in a Phenotypically normal Mother of a Boy with Fragile X Syndrome: Case Report. BMC Med. Genet. 15, 125. 10.1186/s12881-014-0125-2 25421229 PMC4411709

[B44] Mercer T. R. Qureshi I. A. Gokhan S. Dinger M. E. Li G. Mattick J. S. (2010). Long Noncoding RNAs in Neuronal-Glial Fate Specification and Oligodendrocyte Lineage Maturation. BMC Neurosci. 11, 14. 10.1186/1471-2202-11-14 20137068 PMC2829031

[B45] Miller D. T. Adam M. P. Aradhya S. Biesecker L. G. Brothman A. R. Carter N. P. (2010). Consensus Statement: Chromosomal Microarray Is a First-Tier Clinical Diagnostic Test for Individuals with Developmental Disabilities or Congenital Anomalies. Am. J. Hum. Genet. 86, 749–764. 10.1016/j.ajhg.2010.04.006 20466091 PMC2869000

[B46] Monaghan K. G. Lyon E. Spector E. B. (2013). ACMG Standards and Guidelines for Fragile X Testing: a Revision to the Disease-specific Supplements to the Standards and Guidelines for Clinical Genetics Laboratories of the American College of Medical Genetics and Genomics. Genet. Med. 15, 575–586. 10.1038/gim.2013.61 23765048

[B47] Moore S. J. Strain L. Cole G. F. Miedzybrodzka Z. Kelly K. F. Dean J. C. (1999). Fragile X Syndrome with FMR1 and FMR2 Deletion. J. Med. Genet. 36, 565–566. 10.1136/jmg.36.7.565 10424820 PMC1734406

[B48] Myers K. A. van 't Hof F. N. G. Sadleir L. G. Legault G. Simard-Tremblay E. Amor D. J. (2019). Fragile Females: Case Series of Epilepsy in Girls with FMR1 Disruption. Pediatrics 144, e20190599. 10.1542/peds.2019-0599 31439621

[B49] Nagamani S. C. S. Erez A. Probst F. J. Bader P. Evans P. Baker L. A. (2012). Small Genomic Rearrangements Involving FMR1 Support the Importance of its Gene Dosage for normal Neurocognitive Function. Neurogenetics 13, 333–339. 10.1007/s10048-012-0340-y 22890812

[B50] Pastori C. Kapranov P. Penas C. Peschansky V. Volmar C.-H. Sarkaria J. N. (2015). The Bromodomain Protein BRD4 Controls HOTAIR, a Long Noncoding RNA Essential for Glioblastoma Proliferation. Proc. Natl. Acad. Sci. U.S.A. 112, 8326–8331. 10.1073/pnas.1424220112 26111795 PMC4500283

[B51] Pastori C. Peschansky V. J. Barbouth D. Mehta A. Silva J. P. Wahlestedt C. (2014). Comprehensive Analysis of the Transcriptional Landscape of the Human FMR1 Gene Reveals Two New Long Noncoding RNAs Differentially Expressed in Fragile X Syndrome and Fragile X-Associated Tremor/ataxia Syndrome. Hum. Genet. 133, 59–67. 10.1007/s00439-013-1356-6 24005575 PMC3898532

[B52] Peier A. M. McIlwain K. L. Kenneson A. Warren S. T. Paylor R. Nelson D. L. (2000). (Over)Correction of FMR1 Deficiency With YAC Transgenics: Behavioral and Physical Features. Hum. Mol. Genet. 9 (8), 1145–1159. 10.1093/hmg/9.8.1145 10767339

[B53] Penagarikano O. Mulle J. G. Warren S. T. (2007). The Pathophysiology of Fragile X Syndrome. Annu. Rev. Genom. Hum. Genet. 8, 109–129. 10.1146/annurev.genom.8.080706.092249 17477822

[B54] Perry R. B.-T. Hezroni H. Goldrich M. J. Ulitsky I. (2018). Regulation of Neuroregeneration by Long Noncoding RNAs. Mol. Cel 72, 553–567. e555. 10.1016/j.molcel.2018.09.021 PMC6542662 30401432

[B55] Peschansky V. J. Pastori C. Zeier Z. Motti D. Wentzel K. Velmeshev D. (2015). Changes in Expression of the Long Non-coding RNA FMR4 Associate with Altered Gene Expression during Differentiation of Human Neural Precursor Cells. Front. Genet. 6, 263. 10.3389/fgene.2015.00263 26322075 PMC4530595

[B56] Peschansky V. J. Pastori C. Zeier Z. Wentzel K. Velmeshev D. Magistri M. (2016). The Long Non-coding RNA FMR4 Promotes Proliferation of Human Neural Precursor Cells and Epigenetic Regulation of Gene Expression in Trans. Mol. Cell Neurosci. 74, 49–57. 10.1016/j.mcn.2016.03.008 27001315

[B57] Petek E. Kroisel P. M. Schuster M. Zierler H. Wagner K. (1999). Mosaicism in a Fragile X Male Including a De Novo Deletion in theFMR1 Gene. Am. J. Med. Genet. 84, 229–232. 10.1002/(sici)1096-8628(19990528)84:3<229:aid-ajmg13>3.0.co;2-t 10331598

[B58] Probst F. J. Roeder E. R. Enciso V. B. Ou Z. Cooper M. L. Eng P. (2007). Chromosomal Microarray Analysis (CMA) Detects a Large X Chromosome Deletion includingFMR1,FMR2, andIDS in a Female Patient with Mental Retardation. Am. J. Med. Genet. 143A, 1358–1365. 10.1002/ajmg.a.31781 17506108

[B59] Quartier A. Poquet H. Gilbert-Dussardier B. Rossi M. Casteleyn A.-S. Portes V. d. (2017). Intragenic FMR1 Disease-Causing Variants: a Significant Mutational Mechanism Leading to Fragile-X Syndrome. Eur. J. Hum. Genet. 25, 423–431. 10.1038/ejhg.2016.204 28176767 PMC5386424

[B60] Qureshi I. A. Mattick J. S. Mehler M. F. (2010). Long Non-coding RNAs in Nervous System Function and Disease. Brain Res. 1338, 20–35. 10.1016/j.brainres.2010.03.110 20380817 PMC2883659

[B61] Ramaiah M. Tan K. Plank T. D. M. Song H. W. Dumdie J. N. Jones S. (2019). A Micro RNA Cluster in the Fragile‐X Region Expressed during Spermatogenesis Targets FMR 1. EMBO Rep. 20. 10.15252/embr.201846566 PMC6362356 30573526

[B62] Reiss A. L. Dant C. C. (2003). The Behavioral Neurogenetics of Fragile X Syndrome: Analyzing Gene-Brain-Behavior Relationships in Child Developmental Psychopathologies. Dev. Psychopathol. 15, 927–968. 10.1017/s0954579403000464 14984133

[B63] Rogers S. J. Wehner E. A. Hagerman R. (2001). The Behavioral Phenotype in Fragile X: Symptoms of Autism in Very Young Children with Fragile X Syndrome, Idiopathic Autism, and Other Developmental Disorders. J. Develop. Behav. Pediatr. 22, 409–417. 10.1097/00004703-200112000-00008 11773805

[B64] Saldarriaga W. Payán-Gómez C. González-Teshima L. Y. Rosa L. Tassone F. Hagerman R. J. (2020). Double Genetic Hit: Fragile X Syndrome and Partial Deletion of Protein Patched Homolog 1 Antisense as Cause of Severe Autism Spectrum Disorder. J. Dev. Behav. Pediatr. 41, 724–728. 10.1097/dbp.0000000000000850 32947579

[B65] Santhekadur P. K. Kumar D. P. (2020). RISC Assembly and post-transcriptional Gene Regulation in Hepatocellular Carcinoma. Genes Dis. 7, 199–204. 10.1016/j.gendis.2019.09.009 32215289 PMC7083748

[B66] Sitzmann A. F. Hagelstrom R. T. Tassone F. Hagerman R. J. Butler M. G. (2018). Rare FMR1 Gene Mutations Causing Fragile X Syndrome: A Review. Am. J. Med. Genet. 176, 11–18. 10.1002/ajmg.a.38504 29178241 PMC6697153

[B67] Slegtenhorst-Eegdeman K. E. de Rooij D. G. Verhoef-Post M. van de Kant H. J. G. Bakker C. E. Oostra B. A. (1998). Macroorchidism in FMR1 Knockout Mice Is Caused by Increased Sertoli Cell Proliferation during Testicular Development*. Endocrinology 139, 156–162. 10.1210/endo.139.1.5706 9421410

[B68] Smalheiser N. R. Lugli G. (2009). microRNA Regulation of Synaptic Plasticity. Neuromol Med. 11, 133–140. 10.1007/s12017-009-8065-2 PMC3732454 19458942

[B69] Statello L. Guo C. J. Chen L. L. Huarte M. (2021). Gene Regulation by Long Non-coding RNAs and its Biological Functions. Nat. Rev. Mol. Cel Biol. 22, 96–118. 10.1038/s41580-020-00315-9 PMC7754182 33353982

[B70] Symons F. J. Byiers B. J. Raspa M. Bishop E. Bailey D. B. (2010). Self-injurious Behavior and Fragile X Syndrome: Findings from the National Fragile X Survey. Am. J. Intellect. Dev. Disabil. 115, 473–481. 10.1352/1944-7558-115.6.473 20946000

[B71] Tamanini F. Willemsen R. van Unen L. Bontekoe C. Galjaard H. Oostra B. A. (1997). Differential Expression of FMR1, FXR1 and FXR2 Proteins in Human Brain and Testis. Hum. Mol. Genet. 6 (8), 1315–1322. 10.1093/hmg/6.8.1315 9259278

[B72] Tarleton J. Richle R. Schwarsz C. Rao K. Aylsworth A. S. Lachlewicz A. (1993). An Extensive De Novo Deletion Removing FMR1 in a Patient with Mental Retardation and the Fragile X Syndrome Phenotype. Hum. Mol. Genet. 2, 1973–1974. 10.1093/hmg/2.11.1973 8281165

[B73] Tassone F. Hagerman R. J. Chamberlain W. D. Hagerman P. J. (2000). Transcription of the FMR1 Gene in Individuals with Fragile X Syndrome. Am. J. Med. Genet. 97, 195–203. 10.1002/1096-8628(200023)97:3<195:AID-AJMG1037>3.0.CO;2-R 11449488

[B74] Tassone F. Pan R. Amiri K. Taylor A. K. Hagerman P. J. (2008). A Rapid Polymerase Chain Reaction-Based Screening Method for Identification of All Expanded Alleles of the Fragile X (FMR1) Gene in Newborn and High-Risk Populations. J. Mol. Diagn. 10, 43–49. 10.2353/jmoldx.2008.070073 18165273 PMC2175542

[B75] Terracciano A. Chiurazzi P. Neri G. (2005). Fragile X Syndrome. Am. J. Med. Genet. 137c, 32–37. 10.1002/ajmg.c.30062 16010677

[B76] Tsai M.-C. Manor O. Wan Y. Mosammaparast N. Wang J. K. Lan F. (2010). Long Noncoding RNA as Modular Scaffold of Histone Modification Complexes. Science 329, 689–693. 10.1126/science.1192002 20616235 PMC2967777

[B77] Vengoechea J. Parikh A. S. Zhang S. Tassone F. (2012). De Novo microduplication of the FMR1 Gene in a Patient with Developmental Delay, Epilepsy and Hyperactivity. Eur. J. Hum. Genet. 20, 1197–1200. 10.1038/ejhg.2012.78 22549406 PMC3476717

[B78] Verkerk A. J. Pieretti M. Sutcliffe J. S. Fu Y. H. Kuhl D. P. Pizzuti A. (1991). Identification of a Gene (FMR-1) Containing a CGG Repeat Coincident with a Breakpoint Cluster Region Exhibiting Length Variation in Fragile X Syndrome. Cell 65, 905–914. 10.1016/0092-8674(91)90397-h 1710175

[B79] Vittal P. Pandya S. Sharp K. Berry-Kravis E. Zhou L. Ouyang B. (2018). ASFMR1splice Variant. Neurol. Genet. 4, e246. 10.1212/NXG.0000000000000246 30065951 PMC6066363

[B80] Wang Z. Xie Y. Wang Y. Morris D. Wang S. Oliver D. (2020). X‐linked miR‐506 Family miRNAs Promote FMRP Expression in Mouse Spermatogonia. EMBO Rep. 21, e49024. 10.15252/embr.201949024 31808593 PMC6944911

[B81] Wei C.-W. Luo T. Zou S.-S. Wu A.-S. (2018). The Role of Long Noncoding RNAs in Central Nervous System and Neurodegenerative Diseases. Front. Behav. Neurosci. 12, 175. 10.3389/fnbeh.2018.00175 30323747 PMC6172704

[B82] Wöhrle D. Kotzot M. C. Hirst A. Manca B. Korn A. Schmidt G. (1992). A Microdeletion of Less Than 250 Kb, Including the Proximal Part of the FMR-I Gene and the Fragile-X Site, in a Male with the Clinical Phenotype of Fragile-X Syndrome. Am. J. Hum. Genet. 51 (2), 299–306. 1642231 PMC1682683

[B83] Wolff D. J. Gustashaw K. M. Zurcher V. Ko L. White W. Weiss L. (1997). Deletions in Xq26.3-q27.3 Including FMR1 Result in a Severe Phenotype in a Male and Variable Phenotypes in Females Depending upon the X Inactivation Pattern. Hum. Genet. 100, 256–262. 10.1007/s004390050501 9254860

[B84] Yakovlev D. A. Kuznetsova A. A. Fedorova O. S. Kuznetsov N. A. (2017). Search for Modified DNA Sites with the Human Methyl-CpG-Binding Enzyme MBD4. Acta Naturae 9, 88–98. 10.32607/20758251-2017-9-1-88-98 PMC5406665 28461979

[B85] Yu S. Pritchard M. Kremer E. Lynch M. Nancarrow J. Baker E. (1991). Fragile X Genotype Characterized by an Unstable Region of DNA. Science 252, 1179–1181. 10.1126/science.252.5009.1179 2031189

[B86] Zafarullah M. Tang H.-T. Durbin-Johnson B. Fourie E. Hessl D. Rivera S. M. (2020). FMR1 Locus Isoforms: Potential Biomarker Candidates in Fragile X-Associated Tremor/ataxia Syndrome (FXTAS). Sci. Rep. 10, 11099. 10.1038/s41598-020-67946-y 32632326 PMC7338407

[B87] Zhang R. Peng Y. Wang W. Su B. (2007). Rapid Evolution of an X-Linked microRNA Cluster in Primates. Genome Res. 17, 612–617. 10.1101/gr.6146507 17416744 PMC1855169

[B88] Zink A. M. Wohlleber E. Engels H. Rødningen O. K. Ravn K. Heilmann S. (2014). Microdeletions IncludingFMR1in Three Female Patients with Intellectual Disability - Further Delineation of the Phenotype and Expression Studies. Mol. Syndromol. 5, 65–75. 10.1159/000357962 24715853 PMC3977317

