# Supplementary figures and images for: De Novo Large Deletion Leading to Fragile X Syndrome
Source: Front Genet. 2022 May 11;13:884424. doi: 10.3389/fgene.2022.884424 (PMC9130735; doi:10.3389/fgene.2022.884424)

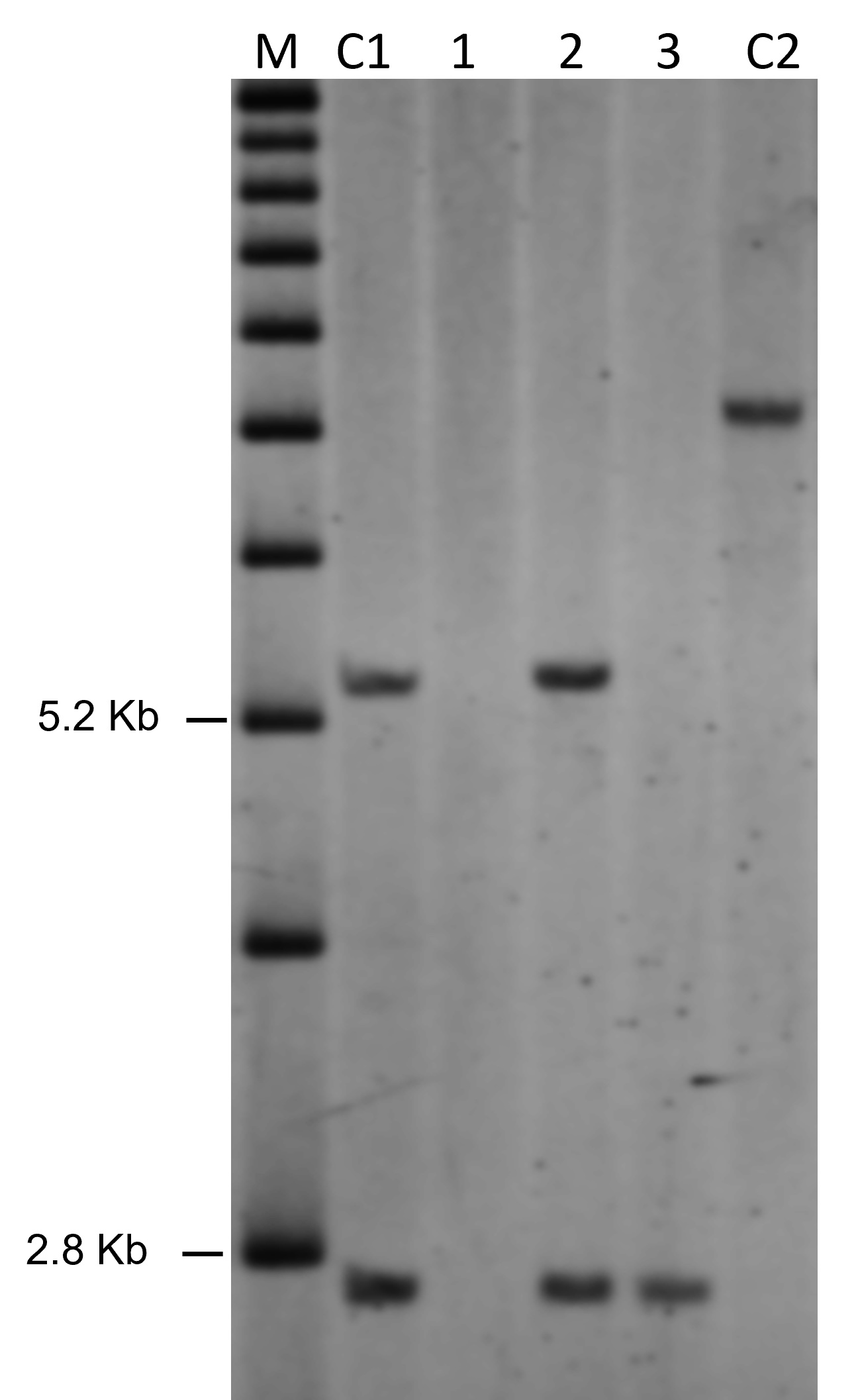

Supplement: Supplementary file 1 [file Image1.JPEG]
